# Dok3 restrains neutrophil production of calprotectin during TLR4 sensing of SARS-CoV-2 spike protein

**DOI:** 10.3389/fimmu.2022.996637

**Published:** 2022-09-12

**Authors:** Jia Tong Loh, Joey Kay Hui Teo, Kong-Peng Lam

**Affiliations:** ^1^ Singapore Immunology Network, Agency for Science, Technology and Research, Singapore, Singapore; ^2^ Department of Microbiology and Immunology, Yong Loo Lin School of Medicine, National University of Singapore, Singapore, Singapore; ^3^ School of Biological Sciences, College of Science, Nanyang Technological University, Singapore, Singapore

**Keywords:** neutrophils, calprotectin, SARS-CoV-2, Dok3, TLR4

## Abstract

Increased neutrophils and elevated level of circulating calprotectin are hallmarks of severe COVID-19 and they contribute to the dysregulated immune responses and cytokine storm in susceptible patients. However, the precise mechanism controlling calprotectin production during SARS-CoV-2 infection remains elusive. In this study, we showed that Dok3 adaptor restrains calprotectin production by neutrophils in response to SARS-CoV-2 spike (S) protein engagement of TLR4. Dok3 recruits SHP-2 to mediate the de-phosphorylation of MyD88 at Y257, thereby attenuating downstream JAK2-STAT3 signaling and calprotectin production. Blocking of TLR4, JAK2 and STAT3 signaling could prevent excessive production of calprotectin by Dok3^-/-^ neutrophils, revealing new targets for potential COVID-19 therapy. As S protein from SARS-CoV-2 Delta and Omicron variants can activate TLR4-driven calprotectin production in Dok3^-/-^ neutrophils, our study suggests that targeting calprotectin production may be an effective strategy to combat severe COVID-19 manifestations associated with these emerging variants.

## Introduction

The Coronavirus disease 2019 (COVID-19) pandemic, caused by severe acute respiratory syndrome coronavirus 2 (SARS-CoV-2), has affected more than 490 million individuals and caused more than 6 million deaths to date (who.int). While majority of the patients are presented with asymptomatic or mild disease, more severe and critical illnesses can arise in a subset of patients due to dysregulated innate immune response, leading to the development of cytokine storm, acute respiratory distress syndrome (ARDS), multiple organ failure, and even death (1). While mass COVID-19 vaccination program is currently underway worldwide, a significant proportion of the population remains unvaccinated, and breakthrough infection is increasingly common in fully vaccinated individuals due to the emergence of new SARS-CoV-2 variants (2–4). Moreover, monoclonal antibody therapies which predominantly target the spike (S) protein of SARS-CoV-2 show diminished potency against newly emerging variants such as the Omicron (5, 6). As such, it is necessary for us to expand and diversify our therapeutic toolbox against SARS-CoV-2 to counteract the ongoing COVID-19 pandemic.

One biomarker which distinguishes mild from severe COVID-19 is serum calprotectin level in infected individuals (7–11). Calprotectin is a stable heterodimer of S100a8 and S100a9 which accumulates in the cytoplasm of neutrophils. During infection, they are released in massive amounts to initiate and amplify inflammatory immune responses, including the production of cytokines and recruitment of leukocytes, through binding to Toll-like receptor (TLR) 4 and receptor for advanced glycation end products (RAGE) (12). However, uncontrolled release of calprotectin by neutrophils can lead to life-threatening systemic inflammation in the host. Calprotectin was reported to be the most abundant immune mediator present in the plasma of severe COVID-19 patients (8), and it correlates strongly with disease severity (7–10). Accordingly, therapeutic agents targeting S100a8-TLR4 or S100a9 can alleviate inflammatory responses and improve survival in preclinical models of SARS-CoV-2 infection (13), thereby emphasizing the clinical significance of calprotectin in COVID-19 pathogenesis (7, 10, 14). However, how SARS-CoV-2 can be sensed by and how calprotectin production can be regulated in neutrophils remain elusive.

Dok3 is an adaptor protein which regulates signaling pathways downstream of various immune receptors. As it lacks intrinsic catalytic activity, it functions primarily as a molecular scaffold to facilitate protein-protein interaction through distinct protein-binding domains (15). Dok3 has been shown to be highly expressed in neutrophils where they play a role in suppressing anti-fungal response downstream of C-type lectin receptors (16). Interestingly, RNA-seq analysis of neutrophils from COVID-19 patients revealed that Dok3 expression is elevated in severe disease cases, suggesting a possible role for Dok3 in SARS-CoV-2 infection in humans (17). However, whether Dok3 is involved in the regulation of calprotectin production in response to SARS-CoV-2 infection remains unexplored.

In this study, we report that Dok3 could suppress the production of calprotectin in neutrophils during SARS-CoV-2 infection when the viral S protein engages TLR4. Dok3 recruits protein tyrosine phosphatase SHP-2 to mediate the de-phosphorylation of MyD88 at Y257, thereby suppressing JAK2-STAT3 signaling axis to prevent excessive calprotectin production by neutrophils. Hence, our study provides novel insight into the mechanism underlying calprotectin production by neutrophils in response to SARS-CoV-2 infection and helps to uncover potential therapeutic signaling molecules which can be targeted to alleviate the massive inflammation in severe COVID-19 patients.

## Results

### Loss of Dok3 enhances calprotectin production by neutrophils in response to SARS-CoV-2 S protein stimulation

**Figure 1:**
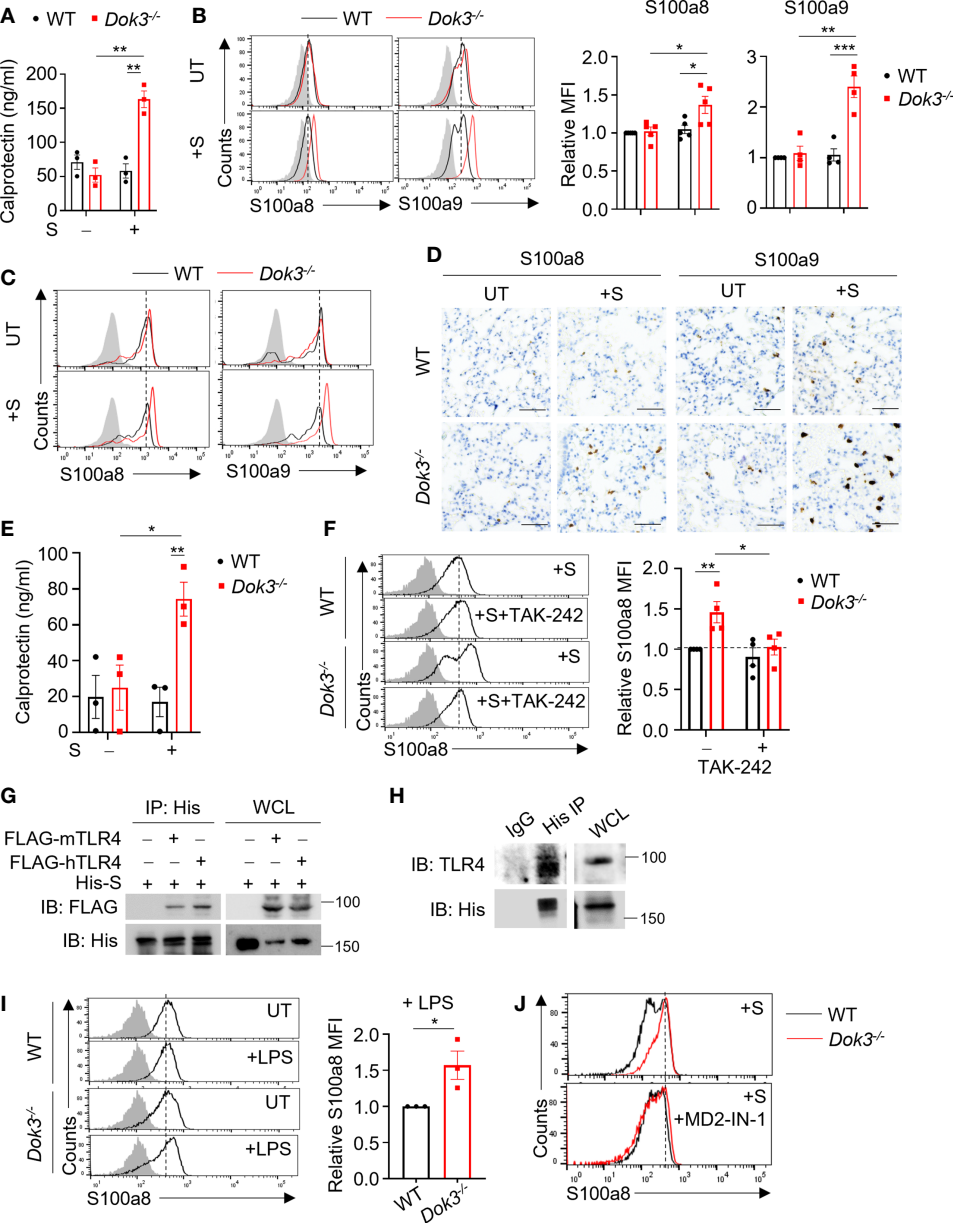
Dok3 suppresses calprotectin production by neutrophils in response to SARS-CoV-2 S protein through a TLR4-dependent pathway. **(A)** WT and *Dok3^-/-^* neutrophils were stimulated with or without S protein for 5h. Calprotectin level in culture medium is measured by ELISA. Data is shown as mean ± S.E.M. (n = 3, 3 independent experiments). **p = 0.002, unpaired two-tailed Student’s t-test. **(B)** Flow cytometric analysis of S100a8 and S100a9 expression in WT and *Dok3^-/-^* neutrophils following 3h stimulation with S protein. Histograms were pre-gated on singlet, Ly6G^+^ cells. Filled histogram represents isotype control. Bar graph depicting MFI of S100a8 or S100a9 fluorescence in *Dok3^-/-^* neutrophils relative to WT neutrophils. Data is shown as mean ± S.E.M. (n = 5, 5 independent experiments). *p = 0.02, 0.03, **p = 0.002, ***p = 0.001, unpaired two-tailed Student’s t-test. (C and D) Lungs were harvested from WT and *Dok3^-/^* ^-^ mice 24h after intranasal instillation of S protein. **(C)** Flow cytometric analysis of S100a8 and S100a9 expression in WT and *Dok3^-/-^* neutrophils following S protein administration. Histograms were pre-gated on singlet, CD45^+^, Ly6G^+^ cells. Filled histogram represents isotype control. **(D)** Lung sections were stained with anti-S100a8 and anti-S100a9 antibodies. Scale bar, 50μm. Data shown are representative of 3 biological replicates. **(E)** Circulating calprotectin levels in WT and *Dok3^-/^* ^-^ mice 24h after intranasal instillation of S protein are measured by ELISA. Data is shown as mean ± S.E.M. (n = 3). *p = 0.03, **p = 0.01, unpaired two-tailed Student’s t-test. **(F)** Flow cytometric analysis of S100a8 expression in WT and *Dok3^-/-^* neutrophils following 3h stimulation with S protein in the presence or absence of TAK-242. Histograms were pre-gated on singlet, Ly6G^+^ cells. Filled histogram represents isotype control. Bar graph depicting MFI of S100a8 fluorescence relative to untreated WT neutrophils. Data is shown as mean ± S.E.M. (n = 4, 4 independent experiments). *p = 0.03, **p = 0.01, unpaired two-tailed Student’s t-test. **(G)** HEK293T cells were transfected with FLAG-tagged mouse (m) or human (h) TLR4. Cell lysates were incubated with His-tagged S protein and immunoprecipitated (IP) with anti-His antibody overnight. Precipitates and whole cell lysates (WCL) were immunoblotted with anti-FLAG and anti-His antibodies. Data shown are representative of 3 independent experiments. **(H)** WT neutrophils were incubated with His-tagged S protein and cell lysates were IP with anti-His or IgG control. Precipitates and WCL were probed for TLR4 and His. Data shown are representative of 3 independent experiments. **(I)** Flow cytometric analysis of S100a8 expression in WT and *Dok3^-/-^* neutrophils following 3h stimulation with or without LPS. Histograms were pre-gated on singlet, Ly6G^+^ cells. Filled histogram represents isotype control. Bar graph depicting MFI of S100a8 fluorescence in *Dok3^-/-^* neutrophils relative to WT neutrophils. Data is shown as mean ± S.E.M. (n = 3, 3 independent experiments). *p = 0.04, unpaired two-tailed Student’s t-test. **(J)** Flow cytometric analysis of S100a8 expression in WT and *Dok3^-/-^* neutrophils following 3h stimulation with S protein in the presence or absence of MD2-IN-1. Histograms were pre-gated on singlet, Ly6G^+^ cells. Filled histogram represents isotype control.

Recent RNA-seq analysis of neutrophils from COVID-19 patients revealed that Dok3 expression is elevated in severe disease cases, suggesting an association with SARS-CoV-2 infection in humans (17). To investigate if Dok3 is involved in calprotectin production in neutrophils during SARS-CoV-2 infection, we stimulated wild-type (WT) and *Dok3^-/-^* neutrophils isolated from mouse bone marrow cells with the ancestral WT SARS-CoV-2 S protein, which has previously been shown to activate both mouse and human immune cells (18), and analyzed for the release of calprotectin (S100a8/9 heterodimer) into the culture medium by ELISA. We observed that treatment with S protein induces a significant increase in calprotectin secretion by *Dok3^-/-^* but not WT neutrophils (**Figure 1A**). Similarly, we found that S100a8 and S100a9 expression were significantly higher in *Dok3^-/-^* as compared to WT neutrophils upon S protein stimulation (**Figure 1B**), suggesting that Dok3 is required to suppress calprotectin production by neutrophils during SARS-CoV-2 infection. Since the S protein can activate immune responses in mouse neutrophils, we further examine the production of calprotectin *in vivo* upon intranasal instillation of S protein in mice. Our histological and flow cytometry analyses revealed higher levels of S100a8 and S100a9 expressed by *Dok3^-/-^* as compared to WT neutrophils in the lungs 24h following S protein administration (**Figures 1C, D**). Moreover, circulating level of calprotectin is significantly increased in *Dok3^-/-^* mice treated with the S protein (**Figure 1E**). Calprotectin are potent initiators and amplifiers of inflammation *via* activation and recruitment of circulating leukocytes. Indeed, we detected increased numbers of S100a8- and S100a9-producing cells, which are defined to be neutrophils *via* flow cytometry, accumulating in the lungs of *Dok3^-/-^* mice (**Figure 1D** and **Supplementary Figure S1**). These reflect a positive feedback loop, in which loss of Dok3 leads to elevated calprotectin production by neutrophils, which subsequently triggers further recruitment of calprotectin-producing neutrophils into the lungs, thereby amplifying aberrant immune responses. Taken together, our data indicate that Dok3 plays a key role in the negative regulation of calprotectin production by neutrophils in response to S protein of SARS-CoV-2.

### TLR4/MD2 is required for sensing of SARS-CoV-2 S protein in neutrophils

The S protein of SARS-CoV-2 has been reported to interact with host receptors TLR2, TLR4 and ACE2 to activate inflammatory immune responses (18–21). To determine which receptor is responsible for sensing S protein upstream of Dok3, we treated WT and *Dok3^-/-^* neutrophils with various inhibitors in the presence of S protein and compare their expression of calprotectin. Treatment with selective TLR4 inhibitor Resatorvid (TAK-242) abolished the enhanced production of S100a8 by *Dok3^-/-^* neutrophils (**Figure 1F**), whereas inhibition of other receptors involved in viral recognition, such as the cell surface ACE2 and TLR2, and the intracellular TLR7 and TLR9, had no effect on S100a8 expression (**Supplementary Figure S2**). These suggest that TLR4 signaling drives calprotectin production in *Dok3^-/-^* neutrophils. To verify if S protein can interact directly with TLR4, we perform co-immunoprecipitation (co-IP) studies with overexpressed TLR4 and SARS-CoV-2 S protein, and observed that both mouse and human TLR4 could interact with the S protein (**Figure 1G**). Endogenous co-IP using lysates from mouse bone marrow neutrophils also revealed interaction between TLR4 and SARS-CoV-2 S protein (**Figure 1H**). Moreover, TLR4 agonist lipopolysaccharide (LPS) also induced a significant increase in S100a8 levels in *Dok3^-/-^* neutrophils (**Figures 1G, I**). Collectively, these revealed that Dok3 inhibits neutrophil production of calprotectin during TLR4 sensing of SARS-CoV-2 S protein.

MD2 is a co-receptor of TLR4 involved in LPS sensing. To determine if MD2 plays a role during TLR4 sensing of SARS-CoV-2 S protein, we treated WT and *Dok3^-/-^* neutrophils with a MD2 inhibitor MD2-IN-1. Here, we observed that MD2 inhibition can abolish the enhanced production of S100a8 by *Dok3^-/-^* neutrophils (**Figure 1J**), suggesting that TLR4/MD2 complex is required for sensing of SARS-CoV-2 S protein in neutrophils.

### Dok3 is not degraded upon TLR4 signaling in neutrophils

Activation of TLR4 signaling by LPS has been reported to induce Dok3 degradation in macrophages (22). To determine if Dok3 is also degraded upon TLR4 activation in neutrophils, we examined Dok3 protein expression in WT neutrophils upon stimulation with S protein or LPS. However, Dok3 expression in TLR4 ligands-stimulated neutrophils remained stable over time, unlike that in LPS-treated macrophages (**Supplementary Figure S3**). This suggest that Dok3 in neutrophils might function in a distinct signaling pathway from macrophages downstream of TLR4.

### Dok3 interacts with MyD88 to mediate its de-phosphorylation on Y257 in response to S protein

**Figure 2:**
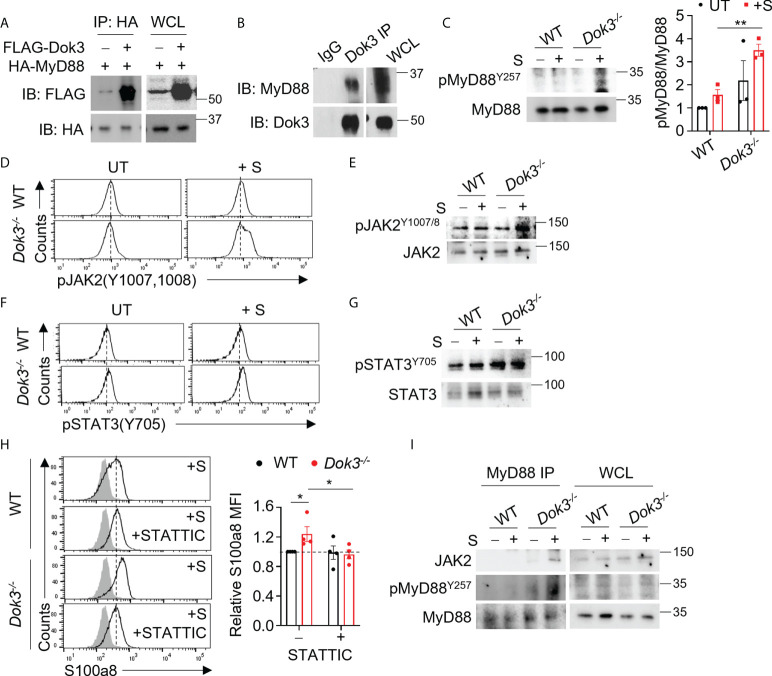
Dok3 mediates de-phosphorylation of MyD88 Y257 and suppression of JAK2-STAT3 signaling in response to SARS-CoV-2 S protein stimulation. **(A)** HEK293T cells were transfected with HA-tagged MyD88 and FLAG-tagged Dok3. Cell lysates were IP with anti-HA antibody. Precipitates and WCL were immunoblotted with anti-HA and anti-FLAG antibodies. Data shown are representative of 3 independent experiments. **(B)** Cell lysates from WT neutrophils were IP with anti-Dok3 or IgG control. Precipitates and WCL were probed for Dok3 and MyD88. Data shown are representative of 3 independent experiments. **(C)** Immunoblot analyses of pMyD88(Y257) and total MyD88 in WT and *Dok3^-/-^* neutrophils treated with or without S protein for 15 mins. Data shown are representative of 3 independent experiments. Bar graph depicts quantification of pMyD88/MyD88 signals from immunoblot. **p = 0.005, unpaired two-tailed Student’s t-test. **(D, F)** Flow cytometric analyses of **(D)** pJAK2(Y1007,1008) and **(F)** pSTAT3(Y705) in WT and *Dok3^-/-^* neutrophils treated with or without S protein for 3h. Histograms were pre-gated on singlet, Ly6G^+^ cells. Data shown are representative of 3 independent experiments (n = 3). **(E, G)**, Immunoblot analyses of **(E)** pJAK2(Y1007,1008), total JAK2, **(G)** pSTAT3(Y705) and total STAT3 in WT and *Dok3^-/-^* neutrophils treated with or without S protein for 15 mins. Data shown are representative of 3 independent experiments. **(H)** Flow cytometric analysis of S100a8 expression in WT and *Dok3^-/-^* neutrophils following 3h stimulation with S protein in the presence or absence of STATTIC. Histograms were pre-gated on singlet, Ly6G^+^ cells. Filled histogram represents isotype control. Bar graph depicting MFI of S100a8 fluorescence relative to untreated WT neutrophils. Data is shown as mean ± S.E.M. (n = 4, 4 independent experiments). *p = 0.05, unpaired two-tailed Student’s t-test. **(I)** Co-IP analysis of WCL from WT and *Dok3^-/-^* neutrophils treated with or without S protein for 15 mins, and IP with anti-MyD88. Precipitates and WCL were probed for JAK2, pMyD88(Y257) and MyD88. Data shown are representative of 3 independent experiments.

MyD88 is a central adaptor protein crucially involved in relaying signals downstream of TLR4 to initiate kinase-dependent signaling during innate immune responses. Recent clinical data suggest that increased expression of MyD88 is associated with the development of severe COVID-19 (23, 24). As such, we postulate that Dok3 may exert an effect on MyD88 during the regulation of TLR4-dependent calprotectin production. To examine possible interactions between Dok3 and MyD88, we overexpressed Dok3 and MyD88 in HEK293T cells, and observed that Dok3 co-IP with MyD88, suggesting an interaction between the two molecules (**Figure 2A**). Similarly, co-IP experiment using cell lysates from mouse bone marrow neutrophils revealed that Dok3 and MyD88 form a complex endogenously (**Figure 2B**). Hence, Dok3 is likely to suppress calprotectin production in neutrophils through a MyD88-dependent mechanism.

Dok3 is a non-catalytic adaptor molecule which controls post-translational regulation by directing various enzymes to their target proteins downstream of immuno-receptors (15, 16). A recent study demonstrated that the activity of MyD88 can be regulated post-translationally *via* phosphorylation of tyrosine residues (25). Indeed, we observed that loss of Dok3 led to elevated phosphorylation of MyD88 at Y257 in *Dok3^-/-^* as compared to WT neutrophils upon stimulation with S protein, indicating that Dok3 mediates the de-phosphorylation of MyD88 in response to SARS-CoV-2 S protein stimulation (**Figure 2C**).

### MyD88 Y257 phosphorylation controls JAK2-STAT3-calprotectin signaling in response to S protein

To delineate the functional role of MyD88 Y257 phosphorylation, we investigated NF-kB and MAPK signaling pathways downstream of MyD88 which are involved in the regulation of inflammatory cytokine production during SARS-CoV-2 infection (24). However, no difference in Erk, NF-kB and p38 activation were observed, and the transcription of inflammatory cytokine genes such as *il1b*, *il6* and *tnfa* were comparable between WT and *Dok3^-/-^* neutrophils (**Supplementary Figures S4A, B**). We next examined JAK2-STAT3 signaling which was previously implicated in calprotectin production in *Dok3^-/-^* colonic neutrophils (26), and detected higher levels of phospho-JAK2 (Y1007/1008) (**Figures 2, D, E**) and its downstream target, phospho-STAT3 (Y705) (**Figures 2F, G**), in *Dok3^-/-^* neutrophils upon stimulation with S protein. On the other hand, phosphorylation of STAT3 at inactivating residue S727 remains unaffected (**Supplementary Figure S4C**). In addition, treatment with a selective STAT3 inhibitor, STATTIC, was able to block S100a8 upregulation in *Dok3^-/-^* neutrophils efficiently in the presence of S protein (**Figure 2H**), thus validating the direct involvement of JAK2-STAT3 pathway in calprotectin production. Previous studies revealed that JAK2 can be activated *via* direct association with the TLR4-MyD88 complex during LPS signaling (27), but how this complex formation is being regulated remains unknown. Since the highly phylogenetically conserved Y^257^KXM motif in MyD88 is a putative SH2 binding site (28), we investigated if phosphorylation of MyD88 at Y257 can regulate JAK2 binding with MyD88. To this end, we performed an endogenous co-IP in neutrophil cell lysates with anti-MyD88 antibody, and found an increased association of JAK2 with MyD88 in *Dok3^-/-^* neutrophils, and these MyD88 molecules were more highly phosphorylated on Y257 than those in WT neutrophils (**Figure 2I**). Collectively, these data indicate that Y257 phosphorylation on MyD88 acts as a molecular switch to turn on downstream JAK2-STAT3 signaling for calprotectin production upon TLR4 recognition of SARS-CoV-2 S protein.

### Dok3 recruits SHP-2 to de-phosphorylate MyD88 in response to S protein

**Figure 3:**
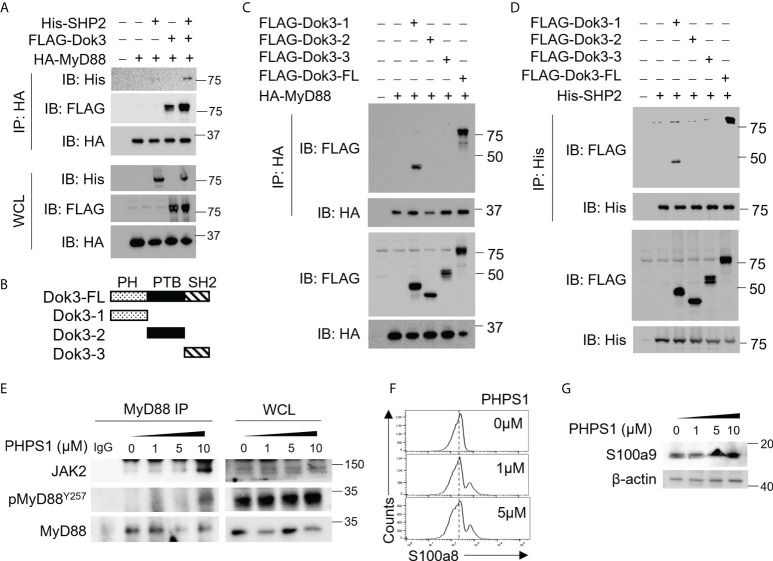
Dok3 recruits SHP-2 to de-phosphorylate MyD88 at Y257 in response to SARS-CoV-2 S protein engagement. **(A)** HEK293T cells were transfected with HA-tagged MyD88, FLAG-tagged Dok3 and His-tagged SHP-2. Cell lysates were IP with anti-HA antibody. Precipitates and WCL were immunoblotted with anti-HA, anti-FLAG and anti-His antibodies. Data shown are representative of 3 independent experiments. **(B)** Diagram depicting Dok3 full-length protein and truncation variants bearing individual PH, PTB, and SH2 domains. **(C)** HEK293T cells were transfected with HA-tagged MyD88 and FLAG-tagged Dok3 or its truncation variants. Cell lysates were IP with anti-HA antibody. Precipitates and WCL were immunoblotted with anti-HA and anti-FLAG antibodies. **(D)** HEK293T cells were transfected with His-tagged SHP-2 and FLAG-tagged Dok3 or its truncation variants. Cell lysates were IP with anti-His antibody. Precipitates and WCL were immunoblotted with anti-His and anti-FLAG antibodies. Data shown are representative of 3 independent experiments. **(E, F)** WT neutrophils were treated with increasing dosages of PHPS1 in the presence of S protein. **(E)** WCL were IP with anti-MyD88. Precipitates and WCL were probed for JAK2, pMyD88(Y257) and MyD88. Data shown are representative of 3 independent experiments. **(F)** Flow cytometric analysis of S100a8 expression in WT neutrophils following PHPS1 treatment. Histograms were pre-gated on singlet, Ly6G^+^ cells. **(G)** Immunoblot analysis of S100a9 expression in WT neutrophils following PHPS1 treatment. Data shown are representative of 3 independent experiments.

Since Dok3 is an adaptor protein which lacks intrinsic catalytic activity, we postulate that it could act as a scaffold to recruit a protein tyrosine phosphatase (PTP) to mediate the de-phosphorylation of MyD88 at Y257. SH2 domain-containing protein tyrosine phosphatase-2 (SHP-2) is a PTP highly expressed in hematopoietic cells. To examine possible interactions among Dok3, SHP-2 and MyD88, we overexpress them in HEK293T cells, and observed that Dok3 interacts with MyD88, while SHP-2 co-IP with MyD88 only in the presence of Dok3 (**Figure 3A**). These data indicate that Dok3 is required to bridge the interaction between SHP-2 and MyD88.

Dok3 is a multidomain adaptor protein containing a N-terminal PH, a central PTB and C-terminal tyrosine-rich SH2 domain (**Figure 3B**). To further characterize the interaction between Dok3, MyD88 and SHP-2, we overexpressed variants of Dok3 bearing specific domains together with MyD88 or SHP-2 and examined their physical associations *via* co-IP experiments. We observed that MyD88 can co-IP with full-length Dok3 as well as the truncated variant bearing the PH domain of Dok3 (**Figure 3C**). Similarly, SHP-2 was observed to bind full-length Dok3 and the variant bearing the PH domain of Dok3 (**Figure 3D**). Hence, Dok3 interacts with both MyD88 and SHP-2 *via* its N-terminal PH domain.

To determine the role of SHP-2 in the de-phosphorylation of MyD88, we treated WT neutrophils with SHP-2 inhibitor PHPS1, and observed a dose-dependent increase in Y257 phosphorylation on MyD88, thus confirming that SHP-2 can act specifically on MyD88 (**Figure 3E**). Moreover, PHPS1 treatment enhances JAK2 association with MyD88 and results in an increased S100a8 and S100a9 production by WT neutrophils in a dose-dependent manner upon stimulation with the S protein (**Figures 3E-G**). We further showed that phosphorylation of SHP-2 on Y580 (**Supplementary Figure S5**), and hence its activity, is not affected by loss of Dok3, implying that altered SHP-2 activity is unlikely to be responsible for enhanced MyD88 Y257 phosphorylation in the absence of Dok3. Taken together, our results suggest that Dok3 is required to recruit SHP-2 to de-phosphorylate Y257 on MyD88 for the suppression of calprotectin production during SARS-CoV-2 infection.

### Fedratinib and Momelotinib suppress S100a8 production by neutrophils in response to SARS-CoV-2 S protein

**Figure 4:**
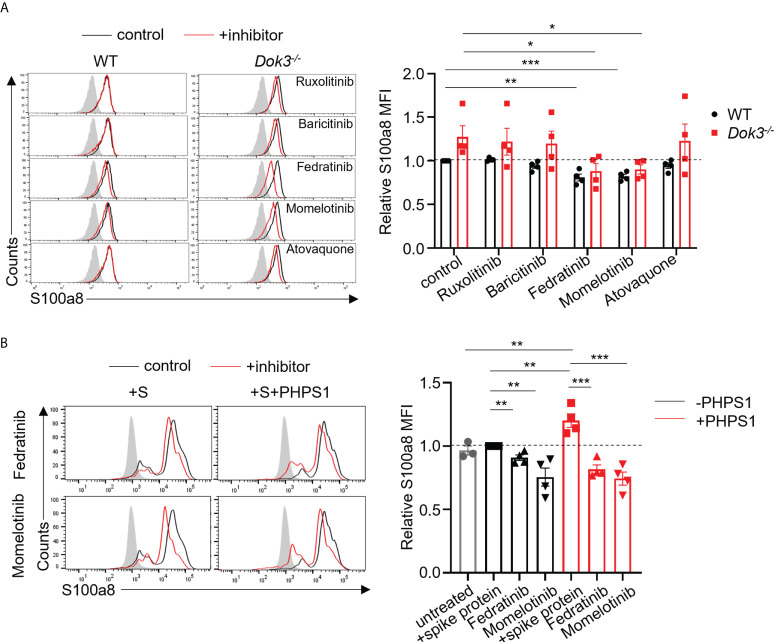
Fedratinib and Momelotinib suppress S100a8 expression in mouse and human neutrophils. **(A)** Flow cytometric analysis of S100a8 expression in WT and *Dok3^-/-^* neutrophils following 3h stimulation with S protein in the presence or absence of indicated inhibitors. Histograms were pre-gated on singlet, Ly6G^+^ cells. Filled histogram represents isotype control. The same control histogram is shown for different inhibitor treatments. Bar graph depicting MFI of S100a8 fluorescence relative to control WT neutrophils. Data is shown as mean ± S.E.M. (n = 4, 4 independent experiments). *p = 0.04, 0.03, **p = 0.003, ***p = 0.0004, unpaired two-tailed Student’s t-test. **(B)** Flow cytometric analysis of S100a8 expression in purified human neutrophils following 3h stimulation with S protein in the presence or absence of indicated inhibitors. Filled histogram represents isotype control. The same control histogram is shown for different inhibitor treatments. Bar graph depicting MFI of S100a8 fluorescence relative to S protein-treated WT neutrophils. Data is shown as mean ± S.E.M. (n = 4, 4 independent experiments). **p = 0.01, 0.006, 0.01, 0.008, ***p = 0.0009, 0.0008, unpaired two-tailed Student’s t-test.

JAK inhibitors have been proposed as therapy for severe COVID-19 patients on the basis that JAKs are important mediators in the cytokine storm (29–31). However, whether these inhibitors can modulate calprotectin levels to improve COVID-19 outcome remains unknown. To this end, we treated WT and *Dok3^-/-^* neutrophils with clinically approved JAK2 and STAT3 inhibitors, including Ruxolitinib, Baricitinib, Fedratinib, Momelotinib and Atovaquone, in the presence of S protein. Among them, we observed that Fedratinib and Momelotinib were able to significantly reduce S100a8 expression by *Dok3^-/-^* neutrophils to WT levels (**Figure 4A**), suggesting that these 2 inhibitors can efficiently block the JAK2-STAT3 signaling pathway downstream of Dok3 to prevent aberrant release of calprotectin in response to SARS-CoV-2 S protein.

To further examine the relevance of our study in human context, we stimulated human neutrophils isolated from peripheral blood of healthy donors with the S protein of SARS-CoV-2. In line with our mouse data, we do not see an induction of S100a8 by human neutrophils in response to S protein (**Figure 4B**). This is also consistent with clinical data which shows that plasma calprotectin levels in COVID-19 patients with mild disease are not elevated as compared to healthy controls (8). Consequently, Fedratinib and Momelotinib treatment only resulted in a minor decrease in S100a8 level (**Figure 4B**). Since our earlier findings indicated that SHP-2 negatively regulates calprotectin production through de-phosphorylating MyD88, we treated human neutrophils with PHPS1 and observed a significant increase in S100a8 production. Moreover, Fedratinib and Momelotinib can block PHPS1-induced S100a8 production (**Figure 4B**), further confirming that JAK2 functions downstream of SHP-2 during calprotectin production. Together, these findings provide a rationale for the use of JAK2 inhibitors Fedratinib and Momelotinib to treat severe COVID-19 patients since they can attenuate calprotectin production in neutrophils.

### SARS-CoV-2 Delta and Omicron variant S proteins bind TLR4 to trigger calprotectin release in the absence of Dok3

**Figure 5:**
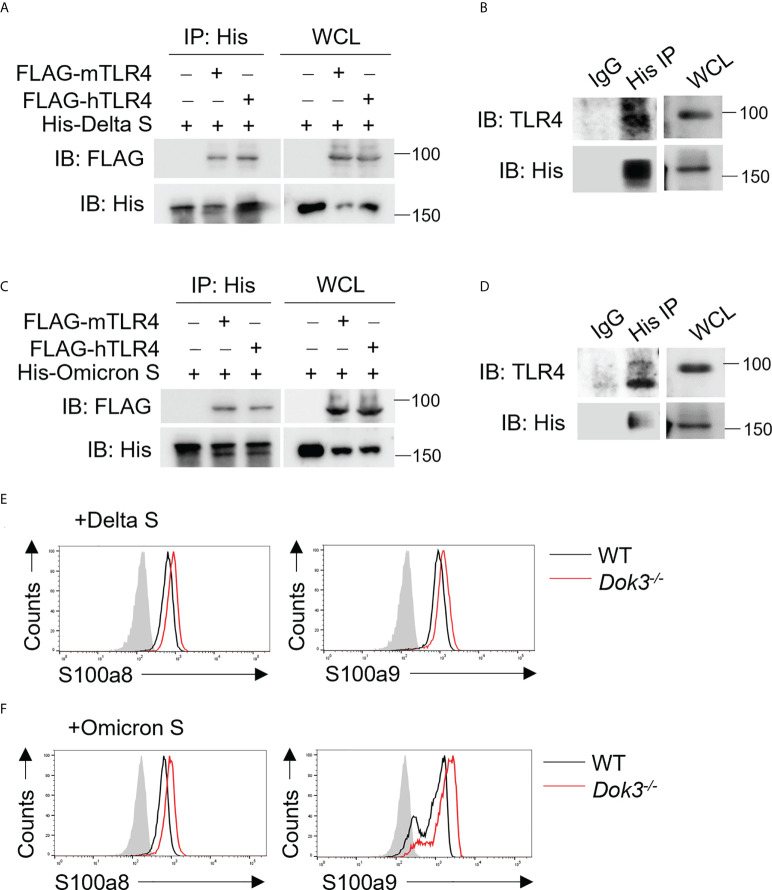
SARS-CoV-2 Delta and Omicron variant S proteins can activate TLR4 signaling. **(A, C)** HEK293T cells were transfected with FLAG-tagged mouse (m) or human (h) TLR4. Cell lysates were incubated with His-tagged S protein derived from **(A)** Delta or **(C)** Omicron variant and IP with anti-His antibody overnight. Precipitates and WCL were immunoblotted with anti-FLAG and anti-His antibodies. Data shown are representative of 3 independent experiments. **(B, D)** WT neutrophils were incubated with His-tagged S protein derived from **(B)** Delta or **(D)** Omicron variant and cell lysates were IP with anti-His or IgG control. Precipitates and WCL were probed for TLR4 and His. Data shown are representative of 3 independent experiments. **(E, F)** Flow cytometric analysis of S100a8 and S100a9 expression in WT and *Dok3^-/-^* neutrophils following 3h stimulation with or without S protein from **(E)** Delta or **(F)** Omicron variant. Histograms were pre-gated on singlet, Ly6G^+^ cells. Filled histogram represents isotype control. Data shown are representative of 3 independent experiments.

The SARS-CoV-2 Delta (B.1.617.2) and Omicron (B.1.1.529) variants harbor distinct amino acid mutations in their S proteins which result in the evasion of host immune responses. As such, we evaluate if these variant S proteins are able to trigger TLR4 signaling in neutrophils by performing co-IP experiments using overexpressed or endogenous TLR4 and Delta or Omicron variant S protein. Interestingly, both Delta and Omicron variant S proteins retained their ability to bind mouse and human TLR4 (**Figures 5A–D**), and this resulted in an enhanced production of S100a8 and S100a9 by *Dok3^-/-^* as compared to WT neutrophils (**Figures 5E, F**). Together, these show that likewise to WT SARS-CoV-2, both Delta and Omicron variant S proteins can activate TLR4-driven calprotectin production in *Dok3^-/-^* neutrophils.

## Discussion

**Figure 6:**
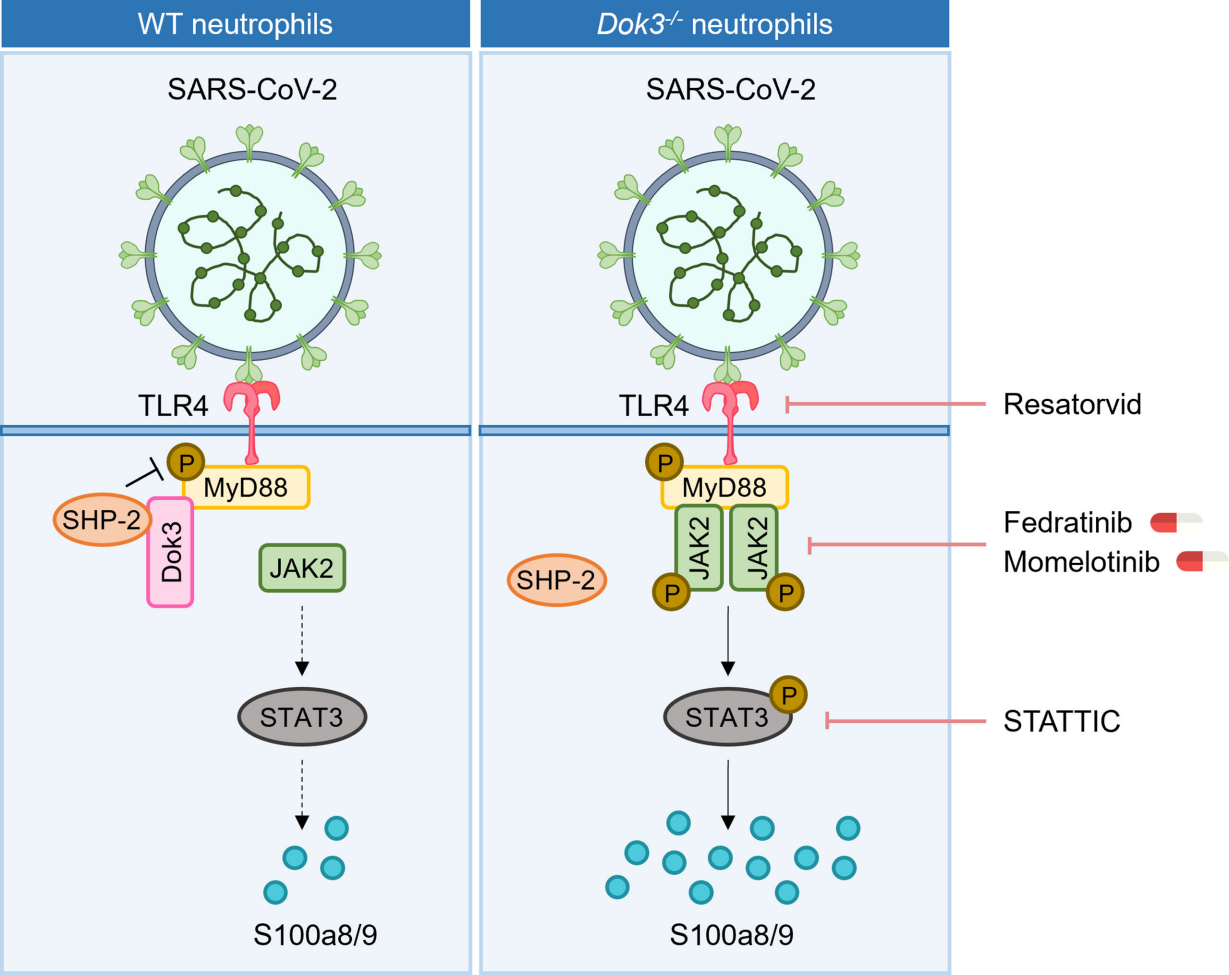
Proposed signaling mechanism underlying Dok3 negative regulation of calprotectin production in neutrophils. Binding of the SARS-CoV-2 S protein to TLR4 triggers phosphorylation of MyD88 on Y257. This activates downstream JAK2-STAT3 signaling cascade to turn on the production of calprotectin. Dok3 negatively regulates this pathway by recruiting SHP-2 to mediate the de-phosphorylation of MyD88, as such preventing excessive release of calprotectin which leads to uncontrolled inflammation associated with severe COVID-19. Treatment with TLR4 inhibitor Resatorvid and STAT3 inhibitor STATTIC, as well as clinically approved JAK inhibitors Fedratinib and Momelotinib, can dampen calprotectin production by neutrophils, revealing potential therapeutic targets to improve disease outcome in severe COVID-19 patients.

During SARS-CoV-2 infection, aberrant production of calprotectin by neutrophils has been linked to the development of severe COVID-19 (8, 9, 11). Our study revealed that Dok3 plays an essential role in restraining neutrophil production of calprotectin upon binding of SARS-CoV-2 S protein to TLR4. In the absence of Dok3, S protein-mediated TLR4 signaling results in the hyper-phosphorylation of Y257 on MyD88, leading to enhanced JAK2-STAT3 signaling and a resultant calprotectin burst in the neutrophils (**Figure 6**).

Calprotectin are stable heterodimers of S100a8 and S100a9 involved in neutrophil-mediated inflammatory processes. Given that calprotectin levels are associated with hyperinflammation and poor clinical outcomes in COVID-19 patients, dissecting the molecular mechanism underlying their regulation will be of great significance and contribute towards the identification of potential therapeutic targets for COVID-19 treatments. In our study, we demonstrated for the first time that calprotectin expression in neutrophils is regulated *via* TLR4 pathway in response to SARS-CoV-2 S protein. In WT cells, S100a8 production is generally suppressed upon TLR4 activation, analogous to clinical studies which show that circulating calprotectin levels remain low in majority of the COVID-19 patients who are asymptomatic or have mild symptoms. In this case, Dok3 recruits SHP-2 to maintain MyD88 Y257 in a de-phosphorylated state, thereby dampening downstream JAK2-STAT3 signaling to prevent excessive release of calprotectin. However, in the absence of Dok3, sensing of S protein by TLR4 promotes increased phosphorylation of MyD88 on Y257. This enhances its association with JAK2 and subsequently turns on downstream JAK2/STAT3 signaling, resulting in hyper-production of calprotectin and an uncontrolled inflammation in the host. Collectively, our findings revealed that Dok3 plays a critical role in the negative regulation of TLR4-MyD88-JAK2-STAT3 axis in neutrophils during SARS-CoV-2 infection for the suppression of calprotectin production, and the increased Dok3 levels detected in neutrophils of severe COVID-19 patients (17) could be a compensatory mechanism to blunt elevated calprotectin levels.

Hyperactivation of neutrophils have been implicated in the immunopathogenesis of COVID-19, in which aberrant NETs formation and enhanced calprotectin secretion are prominent features driving inflammation and tissue damage (32–34). Recent studies demonstrated that SARS-CoV-2 can stimulate neutrophils directly through the ACE2-TMPRSS2 axis to induce release of NETs, thus presenting a potential therapeutic approach to inhibit NETs and its associated devastating complications in severe COVID-19 patients (35). On the other hand, the mechanism governing calprotectin production by neutrophils remains poorly understood. A growing body of evidence suggests that the trimeric S protein of SARS-CoV-2 can interact with TLR4 to activate immune responses (18), and TLR4 signaling has recently been associated with calprotectin production during SARS-CoV-2 infection (13). In our study, we provided compelling evidence that calprotectin production is a tightly regulated process which is triggered upon TLR4 activation by S protein. In the absence of Dok3, calprotectin is robustly upregulated in neutrophils, and this increase is abrogated upon blockade of TLR4 signaling by Resatorvid. We further eliminated the contribution of ACE2 in the induction of calprotectin, since treatment with ACE2 inhibitor does not affect calprotectin production by *Dok3^-/-^* neutrophils, and murine ACE2 harbors differences in amino acids from human ACE2, and hence exhibits poor binding affinity for the S protein (36–38). Thus, our data indicate that SARS-CoV-2 S protein can be sensed directly by TLR4 on neutrophils to regulate calprotectin expression. Consistent with this, it was reported that SARS-CoV-2 infection activates an anti-bacterial like immune response, which was originally proposed to be a result of calprotectin binding to TLR4 (13). Here, our findings suggest that the induction of anti-bacterial pathway genes could be a direct result of interaction between viral S protein and TLR4. We observed that treatment of *Dok3^-/-^* neutrophils with other TLR4 agonists of bacterial origin such as LPS and cecal bacteria similarly induced elevated levels of calprotectin (26). Hence, it is likely that SARS-CoV-2 is sensed by a TLR4-dependent manner analogous to bacteria, to turn on the production of classical anti-bacterial proteins S100a8 and S100a9 in neutrophils.

Currently, no specific treatment is available for COVID-19 patients, and drug repurposing is the most popular strategy adopted to accelerate drug development against a novel emerging pathogen like SARS-CoV-2. As hyperinflammation stemming from dysregulation of the immune system in response to SARS-CoV-2 may culminate into ARDS, thrombosis and multi-organ damage in severe COVID-19 patients, drugs which act to mitigate the cytokine storm are proposed to be beneficial. However, blockade of cytokines such as IL-6 only yield limited clinical success (39–42). On the other hand, calprotectin levels correlate strongly with COVID-19 severity (8, 9, 11), and inhibiting its function can improve disease outcome in preclinical models of SARS-CoV-2 infection (13). Moreover, the sustained release of calprotectin has been proposed to be a key contributor of hyperinflammation and cytokine storm, hence suggesting that targeting calprotectin production is an attractive strategy to alleviate severe COVID-19 manifestations. Here, we provided the first mechanistic study on the regulation of calprotectin production by neutrophils during SARS-CoV-2 infection, and presented several signaling molecules which can be targeted with currently available drugs to limit calprotectin release (**Figure 6**). Firstly, TLR4 inhibitors or antibodies can interfere with the recognition of S protein, thereby preventing the release of calprotectin. In addition, inhibitors of JAK2 and STAT3 can dampen calprotectin production, and we have demonstrated the efficacy of JAK2 inhibitors Fedratinib and Momelotinib in blocking S100a8 release in human neutrophils, thus providing a rationale for the repurposing of these clinically approved drugs for severe COVID-19. However, future preclinical animal models are warranted to investigate the *in vivo* potency and specificity of these drugs for treatment of COVID-19.

SARS-CoV-2 is continuously evolving, and several mutations have been detected in the S protein of Delta and Omicron variants which affected their binding affinities to ACE2 and weakened the neutralizing efficacy of therapeutic and vaccine-elicited antibodies (4–6). Surprisingly, we observed that mutations in S proteins of Delta and Omicron variants did not abolish their binding to TLR4, although we were unable to compare their affinities with respect to that of WT SARS-CoV-2. Given that the Delta and Omicron variants can elicit enhanced calprotectin production by *Dok3^-/-^* neutrophils through a TLR4-dependent manner, it is likely that therapeutic strategies which suppress calprotectin production by neutrophils may still be effective in preventing severe COVID-19 manifestations in patients infected with these variants. This is of paramount importance since extensive S protein mutations on the Omicron variant has severely compromised the efficacy of current vaccines and antibody therapies. As such, an immune-based treatment may be more effective in countering emergent variants as opposed to S protein-directed approaches. Moreover, recent study revealed that aberrant production of calprotectin by neutrophils is a common phenotype observed following coronavirus-induced zoonotic infection (13). Hence, the discovery of promising drug targets to suppress calprotectin production may be a milestone for future treatment of divergent coronavirus-infected patients.

In conclusion, our study identified a key immune signaling pathway involved in the regulation of calprotectin production by neutrophils in response to the S protein of SARS-CoV-2, and further revealed potential therapeutic molecules which can be targeted to dampen the over-expression of calprotectin associated with severe cases of COVID-19.

## Materials and methods

### Mice

C57BL/6 mice were purchased from The Jackson Laboratory. *Dok3^-/-^* mice were generated as described previously (43). *Dok3^-/-^* mice were backcrossed to C57BL/6 mice for more than 10 generations. Male and female mice were used at 8 to 10 weeks of age unless otherwise stated. All mice were maintained under specific pathogen-free conditions at A*STAR Biological Resource Centre (BRC).

### Isolation of mouse neutrophils

Neutrophils were isolated from tibias and femurs of mice using EasySEP Mouse Neutrophil Enrichment Kit (Stem Cell Technologies). Purity of isolated cells was confirmed by flow cytometry. Cells were stimulated with 1μg/ml recombinant SARS-CoV-2 S protein (10549-CV, R&D Systems), B.1.617.2 S protein (10942-CV), B.1.1.529 S protein (SPN,C52Hz, Acro Biosystems) or LPS for indicated time periods.

### Isolation of human neutrophils

Venous blood of healthy human donors was collected and diluted 1:1 in PBS before overlaying on Ficoll-Paque Plus (Cytiva). After density gradient centrifugation, the polymorphonuclear and erythrocyte-rich pellet was collected, and cells were treated with red blood cell lysis buffer for 15 min at room temperature. Cells were subsequently washed with PBS, and neutrophils obtained were resuspended in PBS and rested at 37°C for 1 h.

### Inhibitors

Purified neutrophils were treated with the following inhibitors for 2-4 hours at 37°C: TAK-242 (HY-11109, MedChem Express), STATTIC (sc-202818, Santa Cruz Biotechnology Inc.), Ruxolitinib (HY-50856, MedChem Express), Baricitinib (HY-15315, MedChem Express), Fedratinib (HY-10409, MedChem Express), Momelotinib (HY-10961, MedChem Express), Atovaquone (HY-13832, MedChem Express), MLN-4760, C29 (HY-100461, MedChem Express) and AT791 (HY-124603, MedChem Express).

### Flow cytometry

Cell suspensions were surface labelled with fluorochrome-conjugated antibodies for 10 minutes at 4°C in staining buffer (PBS containing 1% BSA). For phosphoprotein staining, cells were fixed and permeabilized using the Phosflow kit (BD) according to manufacturer’s protocol before staining for 1 hour at room temperature. Data were acquired using LSRII (BD Biosciences) and analyzed using FlowJo software (Tree Star). The following antibodies were used for flow cytometry analysis: anti-CD45 APC/Cy7 (clone 30-F11; BioLegend), anti-Ly6G PE/biotin (clone 1A8; BD, BioLegend), anti-STAT3 (Y705) PE (clone 4/P-STAT3, BD Biosciences), anti-STAT3 (S727) PE (clone 49/P-STAT3, BD Biosciences), anti-pJAK2 (Y1007,1008) Alexa Fluor 647 (clone E132, abcam), anti-Erk1/2 (pT202/Yp204) PE (612593, BD Biosciences), anti-pIKKα/β (S176/180) PE (14938S, Cell Signaling Technology), anti-p-p38 (T180/Y182) FITC (612594, BD Biosciences), anti-S100a8 (clone E4F8V; Cell Signaling Technology), anti-S100a9 (clone D3U8M; Cell Signaling Technology), anti-pSHP2 (Y580) PE (MA5-28045, Invitrogen), goat anti-rabbit IgG (H+L) secondary antibody FITC (Invitrogen).

### Intranasal S protein instillation and histology

20μg of S protein was administered intranasally to WT and *Dok3^-/-^* mice. PBS was used as a negative control. At 24h post-administration, lungs were harvested for flow cytometry analysis or fixed in 4% PFA overnight. For immunohistochemistry, lung sections were stained with anti-S100a8 (clone E4F8V; Cell Signaling Technology), anti-S100a9 (clone D3U8M; Cell Signaling Technology), according to manufacturer’s instructions.

### Calprotectin ELISA

Neutrophils were stimulated with or without S protein in RPMI (Gibco), and cell culture medium was collected after 5 hours. The release of calprotectin into the supernatant was measured by ELISA (ab263885), according to the manufacturer’s instructions.

### Co-IP

For endogenous co-IP, purified neutrophils were stimulated with S protein for 15 minutes before lysis with cell lysis buffer (Cell Signaling Technology) containing protease and phosphatase inhibitors (Cell Signaling Technology). For overexpression studies, HEK293T cells were transfected with the plasmid overnight using lipofectamine (Invitrogen) before lysis with cell lysis buffer. Cell lysates were IP overnight using the indicated antibodies and pulled down using Protein A/G Plus Agarose beads (Santa Cruz Biotechnology Inc.): anti-His (clone H-3; Santa Cruz Biotechnology Inc.), anti-HA (clone F-7; Santa Cruz Biotechnology Inc.), anti-MyD88 (clone E-11; Santa Cruz Biotechnology Inc.), anti-Dok3 (clone H-5; Santa Cruz Biotechnology Inc.), anti-SHP-2 (clone B-1; Santa Cruz Biotechnology Inc.). Precipitates were analyzed by Western blotting according to standard protocol.

### Western blotting

Cells were lysed with cell lysis buffer (Cell Signaling Technology) containing protease and phosphatase inhibitors (Cell Signaling Technology). Cell lysates were analyzed by Western blotting according to standard protocol using the indicated antibodies: anti-pSTAT3 (Y705) (clone D3A7; Cell Signaling Technology), anti-STAT3 (clone 79D7; Cell Signaling Technology), anti-pJAK2 (Y1007/1008) (3771S; Cell Signaling Technology), anti-JAK2 (clone C-10; Santa Cruz Biotechnology Inc.), anti-pMyD88 (Y257) (PA5-64835; Invitrogen), anti-MyD88 (clone E-11; Santa Cruz Biotechnology Inc.), anti-S100a8 (clone E4F8V; Cell Signaling Technology), anti-S100a9 (clone D3U8M; Cell Signaling Technology), anti-Dok3 (clone H-5; Santa Cruz Biotechnology Inc.), anti-β actin (clone C-4; Santa Cruz Biotechnology Inc.), anti-GAPDH (clone 1D4; Santa Cruz Biotechnology Inc.), peroxidase affinipure goat anti-rabbit IgG (H+L) (Jackson ImmunoResearch), m-IgGκ BP-HRP (Santa Cruz Biotechnology Inc.).

### Quantitative PCR

Purified neutrophils were lysed with TRIzol (Life Technologies), and RNA was purified using phenol/chloroform extraction. Complementary DNA was reversed transcribed using RevertAid First Strand cDNA Synthesis Kit (Thermo Fisher Scientific). The following primers were used for real-time PCR using SYBR Green PCR Master Mix (Applied Biosystems): IL6 (forward): GAGGATACCACTCCCAACAGACC; IL6 (reverse): AAGTGCATCATCGTTGTTCATACA; IL1β (forward): CAACCAACAAGTGATATTCTCCATG; IL1β (reverse): GATCCACACTCTCCAGCTGCA; TNFα (forward): GCCTCTTCTCATTCCTGCTTG; TNFα (reverse): CTGATGAGAGGGAGGCCATT; β-actin (forward): AGATGACCCAGATCATGTTTGAGA; β-actin (reverse): CACAGCCTGGATGGCTACGTA.

### Statistics

Figures and statistical analyses were generated using Graphpad Prism software. Mice were allocated to experimental groups based on genotypes and were randomized within their sex- and age-matched groups. No mouse was excluded from the analyses. Unpaired 2-tailed Student’s *t* test was performed. A *P* value of less than 0.05 was considered significant.

### Study approval

All mouse protocols were conducted in accordance with guidelines from and approved by the A*STAR BRC Institutional Animal Care and Use Committee. Human blood was obtained for research with approval from the Centralised Institutional Research Board of the Singapore Health Services in Singapore.

## Data availability statement

The original contributions presented in the study are included in the article/**Supplementary Material**. Further inquiries can be directed to the corresponding authors.

## Ethics statement

The studies involving human participants were reviewed and approved by Centralised Institutional Research Board of the Singapore Health Services in Singapore. Written informed consent for participation was not required for this study in accordance with the national legislation and the institutional requirements. The animal study was reviewed and approved by A*STAR BRC Institutional Animal Care and Use Committee.

## Author contributions

JTL and K-PL conceived and designed the study. JTL and JKHT performed experiments and generated *in vitro* and *in vivo* data which were analyzed and interpreted by JTL. JTL and K-PL wrote the manuscript. All authors reviewed and approved the manuscript.

## Funding

This work is supported by the Singapore Ministry of Health’s National Medical Research Council under its Open-Fund-Individual Research Grant (NMRC/OFIRG19may-0083) to K-PL and JTL, Open Fund-Young Individual Research Grant (NMRC/OFYIRG21nov-0035) to JTL and A*STAR core grant to K-PL.

## Conflict of interest

The authors declare that the research was conducted in the absence of any commercial or financial relationships that could be construed as a potential conflict of interest.

## Publisher’s note

All claims expressed in this article are solely those of the authors and do not necessarily represent those of their affiliated organizations, or those of the publisher, the editors and the reviewers. Any product that may be evaluated in this article, or claim that may be made by its manufacturer, is not guaranteed or endorsed by the publisher.
