# Supplementary material for: Dok3 restrains neutrophil production of calprotectin during TLR4 sensing of SARS-CoV-2 spike protein
Source: Front Immunol. 2022 Sep 12;13:996637. doi: 10.3389/fimmu.2022.996637 (PMC9510782; doi:10.3389/fimmu.2022.996637)
Supplement: Supplementary file 1 [file DataSheet_1.docx]

Supplementary Material

## Supplementary Figures

**
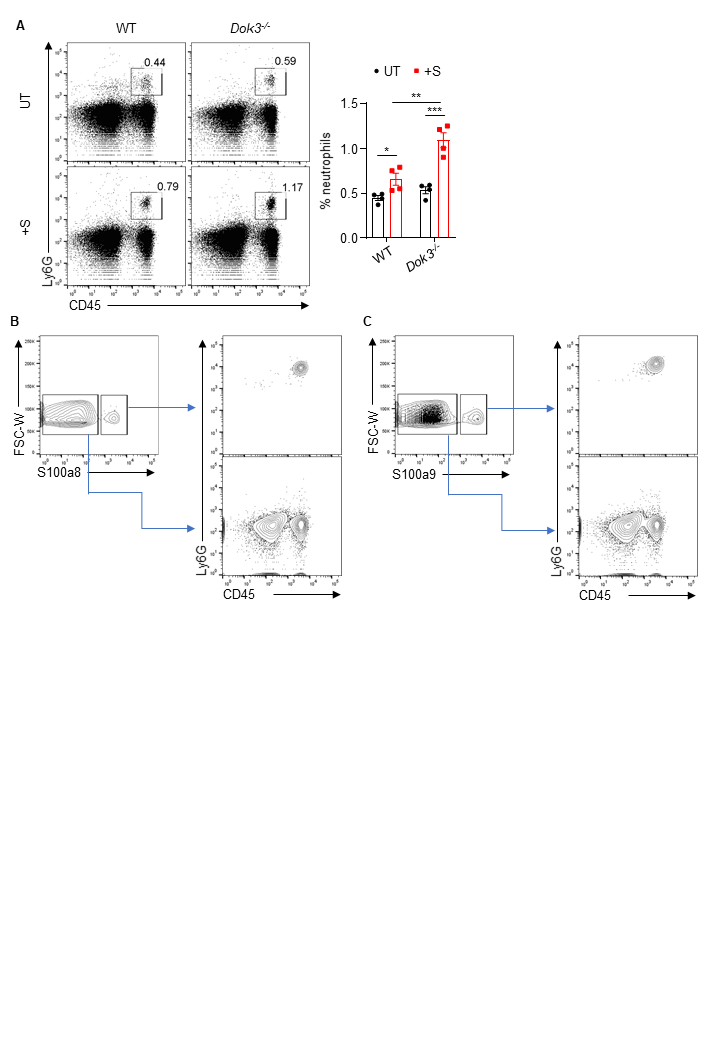
**

**Supplementary Figure 1.** **Increased neutrophils in lungs of *Dok3^-/-^* mice upon S protein instillation. (A)** Flow cytometric analyses of CD45^+^ Ly6G^+^ neutrophils in murine lungs 24h following intranasal S protein administration. *p=0.02, **p=0.006, ***p=0.0009, unpaired two-tailed Student’s t-test. **(B and C)** S100a8- and S100a9-producing cells in the lungs are neutrophils. Flow cytometric analyses of (**B**) S100a8^+^ and (**C**) S100a9^+^ cells in murine lungs 24h following intranasal S protein administration. (**B**) S100a8- or (**C**) S100a9-expressing or non-expressing cells were further analyzed for CD45 and Ly6G expression.

**
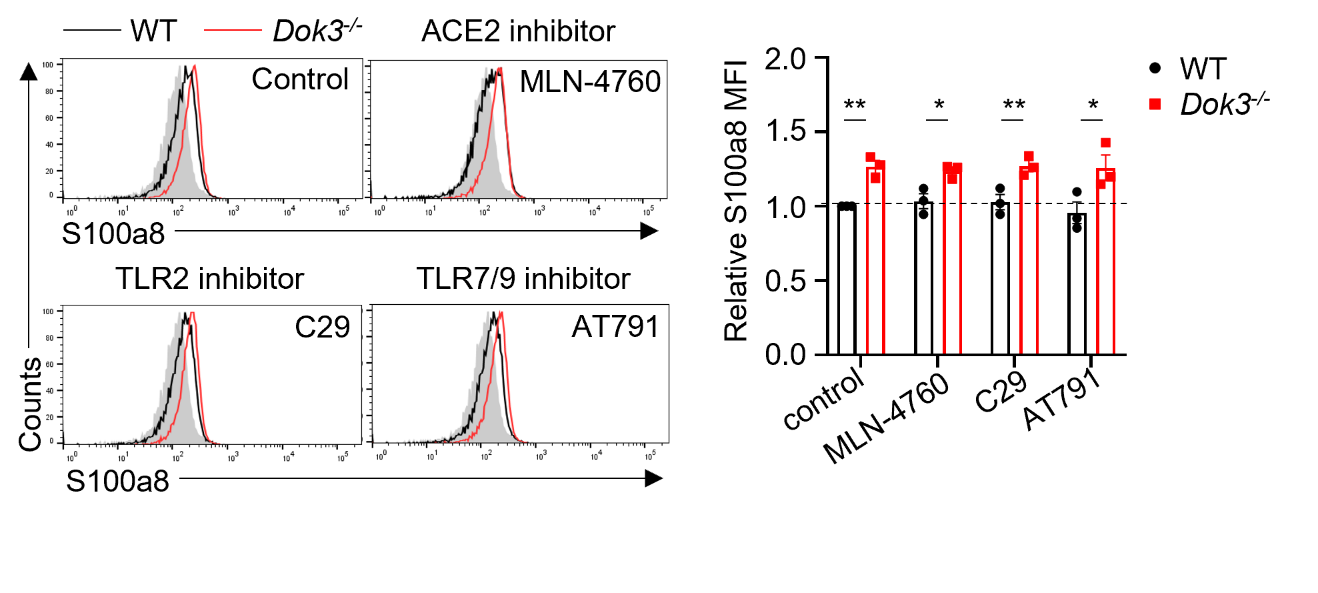
**

**Supplementary Figure 2. ACE2, TLR2, TLR7 and TLR9 are not involved in the sensing of SARS-CoV-2 S protein upstream of Dok3.** Flow cytometric analysis of S100a8 expression in WT and *Dok3^-/-^* neutrophils following 3h stimulation with S protein in the presence or absence of indicated inhibitors. Histograms were pre-gated on singlet, Ly6G^+^ cells. Filled histogram represents isotype control. Bar graph depicting MFI of S100a8 fluorescence relative to untreated WT neutrophils. Data is shown as mean±S.E.M. (n=3, 3 independent experiments). *p=0.02, 0.05, **p=0.003, 0.01, unpaired two-tailed Student’s t-test.

**
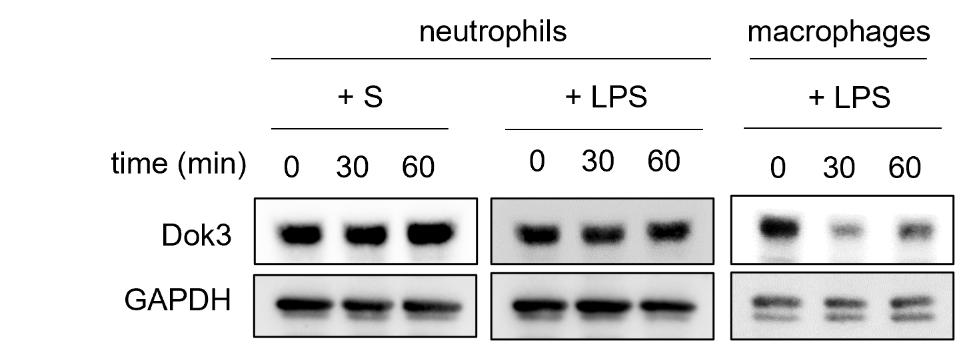
**

**Supplementary Figure 3. Dok3 is not degraded upon TLR4 signaling in neutrophils.** Immunoblot analyses of Dok3 expression in WT neutrophils or macrophages treated with or without S protein or LPS for indicated time periods. GAPDH serves as loading control. Data shown are representative of 3 independent experiments.

**
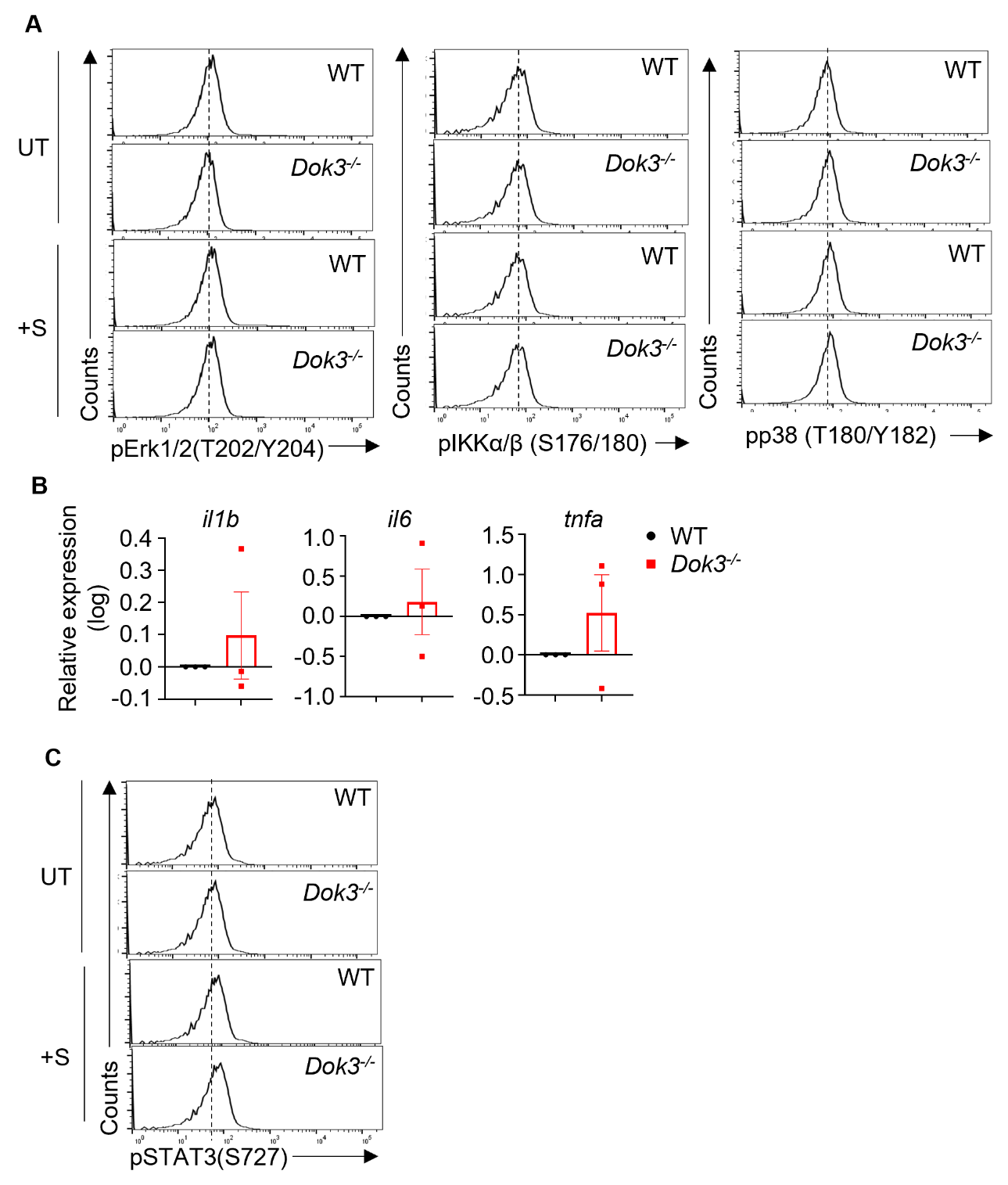
**

**Supplementary Figure 4. Erk, NF-kb and p38 signaling pathways downstream of MyD88 are not affected by Dok3 deficiency. (A)** Flow cytometric analyses of pErk1/2(T202/Y204), pIKKα/β(S176/180) and pp38(T180/Y182) in WT and *Dok3^-/-^* neutrophils treated with or without S protein for 3h. Histograms were pre-gated on singlet, Ly6G^+^ cells. Data shown are representative of 3 independent experiments (n=3). **(B)** RT-qPCR analysis of *il1b, il6* and *tnfa* expression relative to *b-actin* expression in WT and *Dok3^-/-^* neutrophils following 5h stimulation with S protein. Data is shown as mean±S.E.M (n=3, 3 independent experiments). **(C)** Flow cytometric analyses of pSTAT3(S727) in WT and *Dok3^-/-^* neutrophils treated with or without S protein for 3h. Histograms were pre-gated on singlet, Ly6G^+^ cells. Data shown are representative of 3 independent experiments (n=3).

**
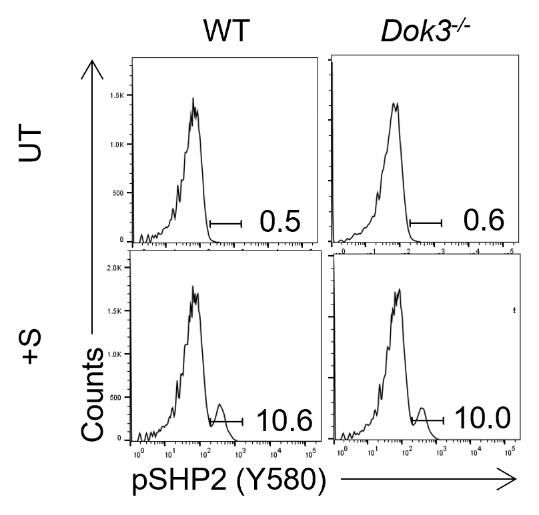
**

**Supplementary Figure 5. Dok3 deficiency does not affect SHP-2 activity.** Flow cytometric analyses of pSHP2(Y580) in WT and *Dok3^-/-^* neutrophils treated with or without S protein for 3h. Histograms were pre-gated on singlet, Ly6G^+^ cells. Data shown are representative of 3 independent experiments (n=3).


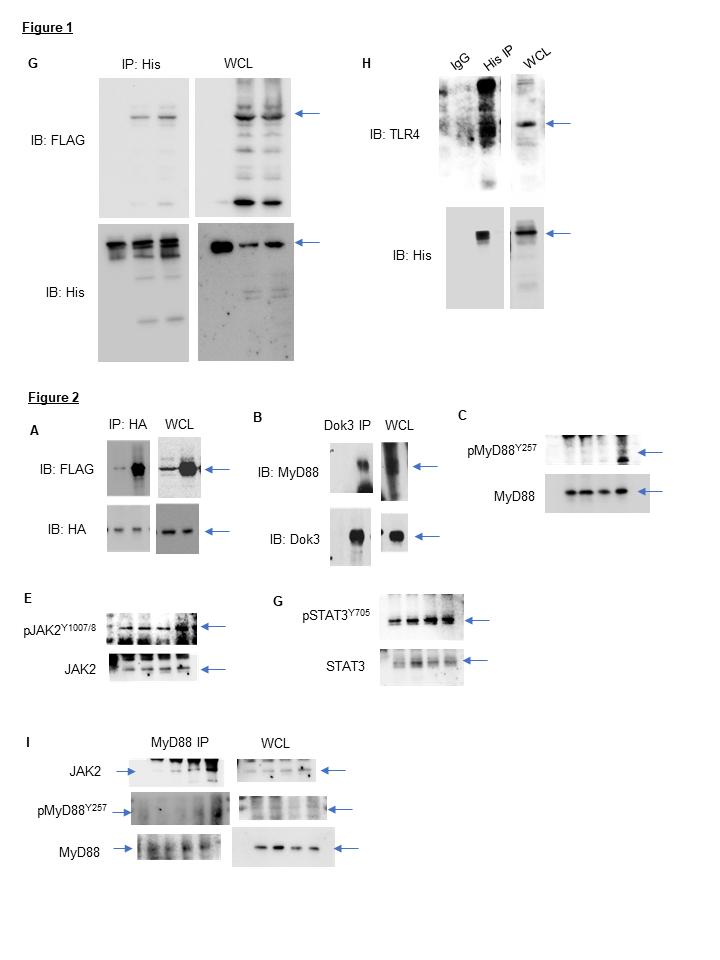

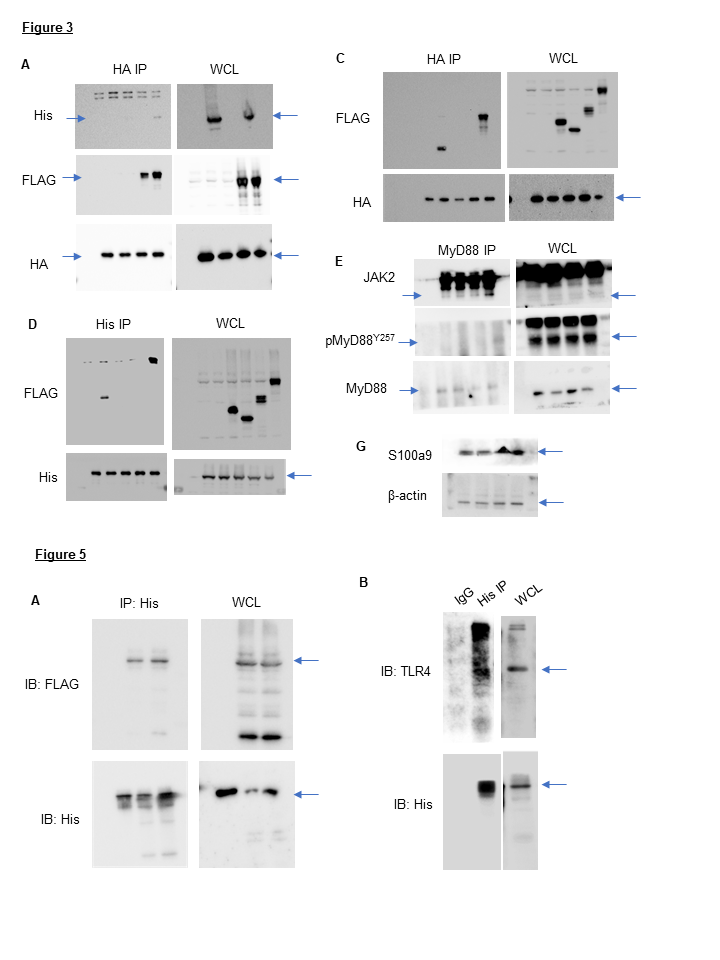


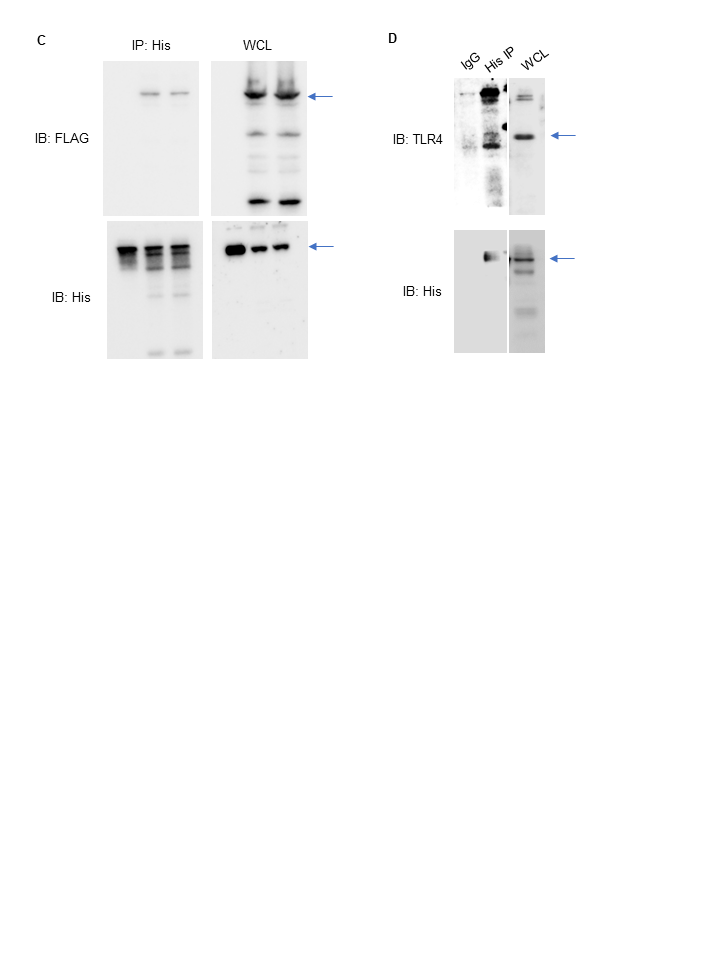


**Supplementary Figure 6. Uncropped images for western blot gel.** Arrows correspond to the bands of interest.
